# Analysis and Modeling of Stress–Strain Curves in Microalloyed Steels Based on a Dislocation Density Evolution Model

**DOI:** 10.3390/ma15196824

**Published:** 2022-10-01

**Authors:** Evelyn Sobotka, Johannes Kreyca, Maria Cecilia Poletti, Erwin Povoden-Karadeniz

**Affiliations:** 1 Christian Doppler Laboratory of Interfaces and Precipitation Engineering, Institute of Materials Science and Technology, TU Wien, 1060 Vienna, Austria; 2 Voestalpine Forschungsservicegesellschaft Donawitz GmbH, 8700 Leoben, Austria; 3 Christian Doppler Laboratory for Design of High-Performance Alloys by Thermomechanical Processing, 8010 Graz, Austria; 4 Institute of Materials Science, Joining and Forming, Graz University of Technology, 8010 Graz, Austria; 5 Institute of Materials Science and Technology, TU Wien, 1060 Vienna, Austria

**Keywords:** computational materials engineering, microalloyed steel, mechanics of materials, flow curve, dislocation density evolution

## Abstract

Microalloyed steels offer a good combination of desirable mechanical properties by fine-tuning grain growth and recrystallization dynamics while keeping the carbon content low for good weldability. In this work, the dislocation density evolution during hot rolling was correlated by materials modeling with flow curves. Single-hit compression tests at different temperatures and strain rates were performed with varying isothermal holding times prior to deformation to achieve different precipitation stages. On the basis of these experimental results, the dislocation density evolution was evaluated using a recently developed semi-empirical state-parameter model implemented in the software MatCalc. The yield stress at the beginning of the deformation *σ*_0_, the initial strain hardening rate *θ*_0_, and the saturation stress *σ*_∞_—as derived from the experimental flow curves and corresponding Kocks plots—were used for the calibration of the model. The applicability for industrial processing of many microalloyed steels was assured by calibration of the model parameters as a function of temperature and strain rate. As a result, it turned out that a single set of empirical equations was sufficient to model all investigated microalloyed steels since the plastic stresses at high temperatures did not depend on the precipitation state.

## 1. Introduction

The energy stored in the material by the distortion produced by dislocations is the main driving force for any kind of recrystallization process. Although this is true for all crystalline materials, recrystallization plays a notable role in microalloyed steels as these are very often thermo-mechanically rolled to achieve a fine-grained structure. The intricate interplay between the deformation-induced dislocation density evolution [1,2]; the precipitation of Ti-, Nb-, and V-carbonitrides [3,4]; the grain growth [5,6]; and the recrystallization [7,8] controls the final mechanical properties [9] of microalloyed (MA) steels. For any model predicting the mechanical properties of an MA steel on a physical basis, a model for the evolution of the dislocation density is thus of utmost importance. For calibration of such a model, experimental values of the dislocation density are necessary. However, it is experimentally challenging to determine the dislocation density evolution in steels after thermo-mechanical treatments using XRD (X-ray diffraction) methods or microstructural observations. The main challenge is the matrix-phase transformation from austenite to ferrite, which irrevocably destroys the dislocation arrangement existing in the austenite before the transformation [10].

An early study by Nabarro et al. [11] summarizes several experimental techniques for the indirect observation of dislocations after deformation in materials that do not experience allotropic phase transformation. The indirect methods are the measurement of density, stored energy [12], surface effects, slip lines, surface markings, etch pits, and XRD [13]. In ferritic steels, dislocation densities were obtained after creep using a combination of electron backscatter diffraction (EBSD) and transmission electron microscopy (TEM) [14]. The resolution of most of these methods is inadequate to provide a detailed insight into the dislocation arrangement. Although XRD can be employed at elevated temperatures [15], the corresponding time resolution needed requires the use of synchrotron sources [16]. Electron channeling contrast imaging (ECCI) allows for quantitative investigations of dislocation structures at the surface of the material [17,18,19]. In situ ECCI measurements are possible but laborious and time consuming. Direct dislocation observation during thermal treatments can be realized via TEM [20,21]; however, intrinsically, it delivers poor statistics.

The modeling of dislocation dynamics has been the topic of several studies in the past [22,23,24,25,26,27]. An analytic model for the dislocation density evolution accounting for dislocation generation, storage, and dynamic recovery mechanisms in polycrystals with solely isotropic material behavior was developed by Hunter and Preston [28]. Zhao et al. [29,30] suggested a model for the overall dislocation density evolution and stress relaxation due to dynamic recovery and recrystallization after hot deformation, but the approach excludes three-dimensional plastic deformation. Buzolin et al. [31] proposed a model to account for the dislocation density evolution during the hot deformation of titanium alloys. This comprehensive approach requires full characterization of the microstructure, and therefore can hardly be applied to materials with allotropic transformation. For crystal plasticity, finite element simulation of stress–strain curves, as well as constitutive models—which often require numerous fitting parameters—are discussed in the literature [30,32,33,34].

The stress–strain curve is a convenient way to gain insight into the dislocation density evolution during plastic deformation. For this, the fundamental relation between the true stress *σ* of a stress–strain curve and the dislocation density ρ of isolated dislocations, as described by the Taylor equation, is used [35,36]:

(1) $$\sigma =\alpha MGb\sqrt{\rho },$$

where *α* is a strengthening coefficient that includes the structural arrangement of the dislocations in a pattern [37] and describes the ratio of the true stress and the dislocation density. *M* represents the Taylor factor; *G* the shear modulus; and *b* the Burgers vector, which describes the magnitude and direction of dislocations. Equation (1) is valid if all dislocations produce the same strain field, such as isolated dislocations, which are not arranged in subgrains or dipoles. On the basis of this relationship, it is possible to derive the dislocation density evolution as a function of strain if the stress–strain curve is known. However, this approach does not allow for a detailed analysis of the dislocation arrangement or the interplay between dislocations and all possible obstacles. It formulates the principle that higher strain hardening results in a higher dislocation density acting as driving force for recrystallization processes. The following chapter defines some general features of stress–strain curves necessary for the application of the used dislocation density evolution model.

### 1.1. Flow Curve Characteristics

Microalloyed steels, similar to the ones used within the scope of this research, were compressed at high temperatures in previous works [38,39]. The main characteristic of the stresses over the strain is the hardening followed by steady-state stress or—if dynamic recrystallization takes place—stress softening. These phenomena are both temperature and time (or strain rate) dependent, evidencing the viscoplastic characteristic of the materials.

**Figure 1:**
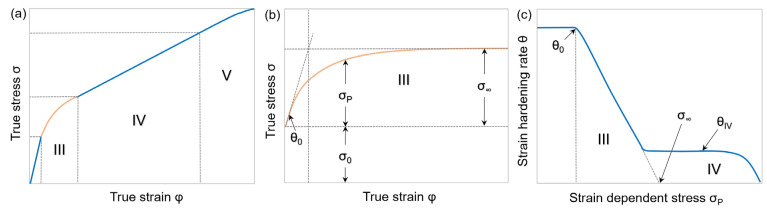
Schematic classification of polycrystal strain hardening behavior into separate stages. (**a**) Entire stress–strain curve with highlighted stage III; (**b**) stage III detail view of flow curve adapted from [43]; (**c**) strain hardening rate θ versus strain-dependent stress *σ*_P_ (Kocks-plot) adapted from [1].

The strain hardening behavior of face-centered cubic metals can be divided into five separate stages [40,41]. The stage III of the stress–strain curves describes the evolution of dislocations by cross-slip movement: the formation and annihilation of subgrains. Stage III prevails in polycrystals such as microalloyed steels and represents the main focus of this work (Figure 1a). At this stage—which is very sensitive to temperature and strain rate—the true stress *σ* rises while the strain hardening rate *θ* decreases. The parameter *θ* is defined as *dσ*/*dφ*, with *φ* describing the true strain. The true stress *σ* of the stress–strain curves during deformation is the result of the contributions of the slightly strain-dependent initial yield stress *σ*_0_ and the strongly strain-dependent plastic stress *σ*_P_ (Figure 1b), the latter being controlled by the dislocation density development. Figure 1c shows that *θ* plotted against *σ*_P_—known as Kocks-plot—reveals a linear relationship between the strain hardening rate *θ*_0_ at the beginning and the saturation stress *σ*_∞_ at the end of stage III. The strain hardening rate reaches a plateau at *θ*_IV_ in stage IV [1,42].

In general, any stress–strain curve (stage III) is thus adequately represented by the three parameters *σ*_0_, *σ*_∞_, and *θ*_0_. With *σ* = *σ*_P_ + *σ*_0_, the dislocation density can then be determined by employing Equation (1). Once the physical parameters *σ*_0_, *σ*_P_, and *θ*_0_ are derived from experimentally determined stress–strain curves, it is possible to apply the model of Kocks and Mecking [1,42,44] for the accurate reproduction of the dislocation density evolution necessary to deliver the measured shape of the stress–strain curve.

### 1.2. Carbonitrides in Microalloyed Steel

The present work focuses on the effect of the microalloying elements Ti, Nb, and V, as well as their carbonitride phases on plastic deformation at elevated temperatures. The main difference between the effect of Ti, Nb, and V microalloying has its origin in their solubility limits within the steel matrix. In general, Ti carbonitrides remain stable up to a much higher temperatures than Nb(C,N) and especially V(C,N). Therefore, the alloying of Ti is most effective for pinning grain boundaries at high temperatures [3,45]. Nb carbonitrides form at temperatures just above the recrystallization temperature, and their precipitation process is quite slow [46,47]. However, the number of nucleation sites, as well as diffusion-controlled processes, can be enhanced by plastic deformation. The precipitation of Nb(C,N) can additionally reduce the high angle grain boundary mobility and consequently delay the static recrystallization process that would otherwise start immediately after the deformation [48,49]. V carbonitrides have a much higher solubility in austenite than in ferrite and will therefore precipitate in a finely dispersed manner during or just after the austenite to ferrite transformation. V is often added to enhance the strength by precipitation strengthening [50,51].

The motivation of this publication is to evaluate the evolution of dislocation densities via simple equations based on the Kocks–Mecking approach for future implementation in deformation-affected precipitation kinetics in combination with recrystallization.

## 2. Model Approach

The single-state parameter in the applied model is the dislocation density. Modeling plastic stress as a function of the dislocation density in contrast to modeling it as a function of strain comes with several advantages. Firstly, the dislocation density is an internal variable of the system that can be directly measured via TEM [20,21] or by in situ X-ray diffraction [52]. Only in situ measurements are possible in the case of materials with allotropic transformation such as the here-studied steels. In our work, the dislocation density was calculated via the presented equations and compared to the state-of-the-art measurements. Strain is only defined as related to a certain deformation history and cannot be measured a posteriori. Secondly, it allows for the connection with recrystallization models in which the driving force is commonly described as a function of the dislocation density.

### 2.1. Dislocation Density Model

The microstructural state parameter-based model, describing the strain-induced dislocation density evolution by Kocks and Mecking [1,42,44], is employed for the computational reproduction of experimentally derived flow curves:

(2) $$\frac{d\rho }{d\varphi }=\frac{d\rho^{+}}{d\varphi }-\frac{d\rho^{-}}{d\varphi }=k_{1}\sqrt{\rho }-k_{2}\rho ,$$

where $d\rho^{+}/d\varphi =k_{1}\sqrt{\rho }=(M/bP)\sqrt{\rho }$, representing the dislocation storage term, accounts for the generation of dislocations by plastic deformation. The proportionality constant *P* correlates the mean free path of mobile dislocations *L* and the dislocation density *ρ* via $P=L\sqrt{\rho }$. The term $d\rho^{-}/d\varphi =k_{2}\rho$ describes the dislocation annihilation due to dynamic recovery [53].

The combination of the Taylor Equation (1) and the Kocks–Mecking Equation (2) allows for the expression of the development of the strain-hardening rate by the following linear equation:

(3) $$\frac{d\sigma_{P}}{d\varphi }=\frac{k_{1}}{2}\alpha MGb-\frac{k_{2}}{2}\sigma_{P}.$$

As true stress and dislocation density are related via the Taylor Equation (1), a strain-dependent evolution of *ρ* based on the Kocks–Mecking model, Equation (2), allows for the description of stress–strain diagrams. The shape of the stress–strain curves can be characterized by Equation (3) after the determination of *k*_1_ and *k*_2_ using the initial strain hardening rate *θ*_0_ and the saturation stress *σ*_∞_ [54]:

(4) $$k_{1}=\frac{2\theta_{0}}{\alpha MGb},$$

(5) $$k_{2}=\frac{2\theta_{0}}{\sigma_{\infty }}.$$

On the basis of the research of Kocks and Mecking [1,42,44], the mean-field ABC-model [55] was developed and implemented in MatCalc to calculate the dislocation density evolution during deformation.

### 2.2. ABC-Model

The ABC-model is a modified Kocks–Mecking model extended by an additional third term that accounts for the impact of high temperatures on the dislocation movement. The terms A, B, and C control the three mechanisms: generation of dislocations, dynamic recovery, and static recovery, respectively. Equation (2) is then written as follows [56,57]:

(6) $$\frac{\partial \rho }{\partial \varphi }=\frac{M}{bA}\sqrt{\rho }-2BM\frac{d_{\mathrm{crit}}}{b}\rho -2CD_{d}\frac{Gb^{3}}{\dot{\varphi }kT}\left(\rho^{2}-\rho_{\mathrm{eq}}^{2}\right),$$

where *d*_crit_ describes the critical annihilation distance between two dislocations; *D*_d_ is the diffusion coefficient; $\dot{\varphi }$ is the strain rate; *k* is the Boltzmann constant; *T* is the temperature; and *ρ*_eq_ is the initial dislocation density, which is reached at annealed material state (equilibrium dislocation density).

The parameter *A* mainly describes the free path of moving dislocations until their impediment by immobile dislocations [55]:

(7) $$A=\frac{\alpha GbM^{2}}{2b\theta_{0}}.$$

The term of the dislocation annihilation coefficient *B* is related to the inverse probability of a dislocation to annihilate on the condition that a second dislocation with a critical diameter is present. *C* is the dislocation annihilation parameter for static recovery. At low temperatures, the term C of Equation (6) can be neglected. The value of *B* can therefore be calculated via the following equation [55]:

(8) $$B=\frac{b\theta_{0}}{\sigma_{\infty }d_{\mathrm{crit}}M}.$$

At elevated temperatures, the term *B* of Equation (6) is neglected, and the parameter *C* can be formulated as [55]

(9) $$C=\frac{{\left(\alpha GbM\right)}^{2}}{{\left(\sigma_{\infty }\right)}^{3}}\frac{\theta_{0}\dot{\varphi }kT}{D_{d}Gb^{3}}.$$

The following equations describe additional parameters needed for the ABC-model according to [55]:

(10) $$d_{\mathrm{crit}}=\frac{Gb^{4}}{2\pi \left(1-\nu \right)Q_{\mathrm{vac}}},$$

(11) $$D_{d}=D_{d0}\exp\left(-\frac{Q_{d}}{kT}\right),$$

where *ν* represents the Poisson’s ratio, *Q*_vac_ is the vacancy formation energy in the austenite phase, *D*_d0_ is the pre-exponential factor for pipe diffusion in the austenite phase, and *Q*_d_ is the activation energy for pipe diffusion in the austenite phase.

### 2.3. MatCalc Strength Modeling

Within the utilized simulation software MatCalc [58], the total yield strength *σ* of the precipitation matrix is divided into a thermal part (*σ*_0_) and an athermal part (*σ*_P_), which does not need thermal activation [56]:

(12) $$\sigma =\sigma_{P}+\sigma_{0},$$

(13) $$\sigma_{P}=\sigma_{\mathrm{DIS}}\cdot \left(1-X_{a}\right),$$

(14) $$\frac{1}{\sigma_{0}}=\frac{1}{\sigma_{0}^{\mathrm{lt}}}+\frac{1}{\sigma_{0}^{\mathrm{ht}}},$$

where *σ*_DIS_ is the dislocation yield strength contribution, and *X*_a_ is the recrystallized fraction of the austenite phase. The thermally activated *σ*_0_^lt^ and *σ*_0_^ht^ account for deformation at low as well as high temperatures, respectively [56]:

(15) $$\sigma_{0}^{\mathrm{lt}}=\hat{\sigma }{\left(\frac{\dot{\varphi_{0}}}{\dot{\varphi }}\right)}^{-kT/\Delta F_{0}^{\mathrm{lt}}},$$

(16) $$\sigma_{0}^{\mathrm{ht}}={\left(\hat{\sigma }\frac{\dot{\varphi }kT{\left(\alpha bGM\right)}^{2}}{2bc\Delta F_{0}^{\mathrm{ht}}\exp(-\Delta F_{0}^{\mathrm{ht}}/kT)}\right)}^{1/3},$$

where $\hat{\sigma }$ is the mechanical threshold, $\dot{\varphi }$ is the strain rate, $\dot{\varphi_{0}}$ is a constant pre-exponential factor, $\Delta F_{0}^{\mathrm{lt}}$ is the energy for a dislocation to overcome an obstacle without thermal activation, *c* is the velocity of sound, and $\Delta F_{0}^{\mathrm{ht}}$ is the energy that must be supplied in the absence of any thermal activation [56]. The value of $\hat{\sigma }$ is calculated as follows:

(17) $$\hat{\sigma }=\sigma_{\mathrm{BASIC}}+\sigma_{\mathrm{SS}}+\sigma_{\mathrm{GS}}+\sigma_{\mathrm{PREC}},$$

where *σ*_BASIC_ is the basic yield strength of the pure iron matrix, *σ*_SS_ is the solid solution strength contribution based on Labusch’s findings [59], *σ*_GS_ is the grain boundary strength contribution according to Hall and Petch [60,61], and *σ*_PREC_ is the total strength contribution of precipitates at room temperature. Here, precipitation hardening is controlled by two mechanisms either shearing or bypassing (Orowan) [62]:

(18) $$\tau_{\mathrm{PREC}}={\left[{\left(\sum \tau_{i,\mathrm{sh}}^{m_{\mathrm{sh}}}\right)}^{m_{\mathrm{tot}}/m_{\mathrm{sh}}}+{\left(\sum \tau_{i,\mathrm{nsh}}^{m_{\mathrm{nsh}}}\right)}^{m_{\mathrm{tot}}/m_{\mathrm{nsh}}}\right]}^{1/m_{\mathrm{tot}}},$$

(19) $$\sigma_{\mathrm{PREC}}=M\cdot \tau_{\mathrm{PREC}},$$

where $\tau_{\mathrm{PREC}}$ is the total precipitate shear stress, calculated via the shear stress contributions for shearable $\tau_{\mathrm{sh}}$ and non-shearable $\tau_{\mathrm{nsh}}$ precipitates of type *i* [62]. The value of the exponent *m*_sh/nsh_ is 1.4, while *m*_tot_ is selected as 1.8.

For the additional examination of the thermally activated stress contribution solely affected by precipitates (*σ*_PREC_TA_), the limiting case for the mechanical threshold $\hat{\sigma }=\sigma_{\mathrm{PREC}}$ can be assumed. In this instance, the basic yield strength, the solid solution strength, and the grain boundary strength have to be excluded when calculating $\hat{\sigma }$. This assumption is feasible, as the single strength contributions are generally summed up additively without interconnected correlations regarding the calculation of $\hat{\sigma }$. Therefore, via the combination of Equations (14)–(16), thermally activated precipitation hardening is calculated:

(20) $$\frac{1}{\sigma_{\mathrm{PREC}\_\mathrm{TA}}}=\frac{1}{\sigma_{0}^{\mathrm{lt}}{}_{\left(\hat{\sigma }=\sigma_{\mathrm{PREC}}\right)}}+\frac{1}{\sigma_{0}^{\mathrm{ht}}{}_{\left(\hat{\sigma }=\sigma_{\mathrm{PREC}}\right)}}.$$

## 3. Materials and Methods

Five different grades of microalloyed steel with low carbon content were investigated. The sample material was taken from industry. Table 1 lists their chemical compositions.

Steel grades S3–S5 were used to investigate the strain-hardening behavior of typical microalloyed steels. The material S5 is primarily used to analyze the impact of different holding times on the precipitate evolution and therefore on the resulting flow curves. S5 contains higher amounts of the microalloying elements Nb and V, while S3 and S4 only contain V or Nb, respectively. S1 and S2 served as reference alloys. S1 comprises a notable higher amount of Cr and Ti in comparison to the other microalloyed materials S3–S5, and S2 contains only minor amounts of microalloying elements.

### 3.1. Simulation

The focus of this work was to find any influence of the precipitates on the deformation process at elevated temperatures, i.e., to answer the question of how far precipitates alter the stress–strain curves and thus the dislocation density evolution of microalloyed steels during hot deformation.

#### 3.1.1. MatCalc Parameters for the Simulation Setup

For the predictive strain hardening in combination with precipitation kinetics based on thermodynamic [63] and diffusion mobility [64] databases implemented in the mean-field microstructure modeling software package MatCalc [58], sensible input parameters for the dislocation density evolution are required. For the determination of A, B, and C the experimentally determined *σ*_0_, *σ*_∞_, and *θ*_0_ and Equations (7)–(9) were employed for the implemented ABC-model within MatCalc. For the precipitation simulation, the nucleation sites of (Ti,Nb,V)(C,N) carbonitrides were selected as dislocations [65]. The regular solution critical temperature *T*_crit_ of the examined particles (TiCN: 4000 °C, NbCN: 3100 °C, VCN: 2600 °C) was estimated from the miscibility gaps of the corresponding binary phase diagrams. Simulations of the present study were performed using the values in Table 2 and a strengthening coefficient *α* of 0.12. This factor depends on the dislocation pattern heterogeneity and decreases at high deformation [37,66]. For the calculated values of the critical annihilation distance between two dislocations *d*_crit_ and the diffusion coefficient *D*_d_, Equations (10) and (11) were used, respectively. Activation energy for pipe diffusion in the austenite phase of *Q*_d_ = 185∙10^3^ [Jmol^−1^] and the pre-exponential factor of *D*_d0_ = 4.5∙10^−5^ [m^2^s^−1^] was used for the calculation of the diffusion coefficient *D*_d_ [67].

#### 3.1.2. Preparatory Equilibrium Calculations

**Figure 2:**
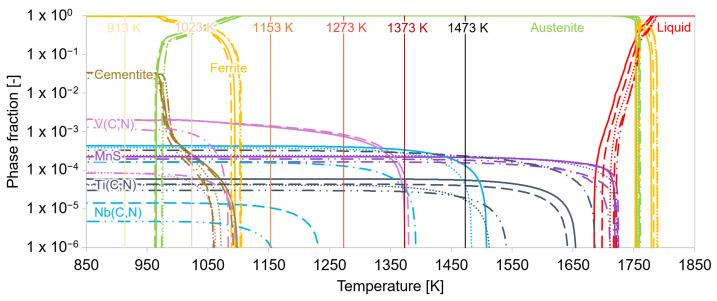
Equilibrium calculations of MA steels S5–S1. The vertical lines indicate the individual deformation temperatures.

Equilibrium calculations based on the Calphad method [71] were conducted to obtain the relevant temperature intervals where precipitation processes take place. The temperatures at which (Ti/Nb/V)(C,N) carbonitrides are present were identified via equilibrium calculations using the software MatCalc. In the stepped simulation, the phase fractions were calculated as a function of a specific temperature for a particular alloy composition. Figure 2 compares the equilibrium phase fractions of all five examined steel grades. The amount of precipitated carbonitrides clearly increased at lower temperatures.

The solubility limits of the different microalloying carbonitrides strongly depend on the composition of the individual alloy, much more than that of the matrix phase. On the basis of these results, the test temperatures for the deformation experiments were selected. Two temperatures deserve special consideration, i.e., the annealing temperatures at 1553 K and the test temperature of 1153 K. The solution annealing temperature of 1553 K for the experiments was chosen to be above the solubility limit of the Nb(C,N) phases. The temperature of 1153 K was chosen for deformation tests with different prior annealing times. This temperature is well in the stable austenite phase but low enough for V(C,N) and Nb(C,N) to precipitate in the alloyed materials. Varied annealing times were supposed to result in different size distributions of the carbonitrides.

### 3.2. Experiments

**Figure 3:**
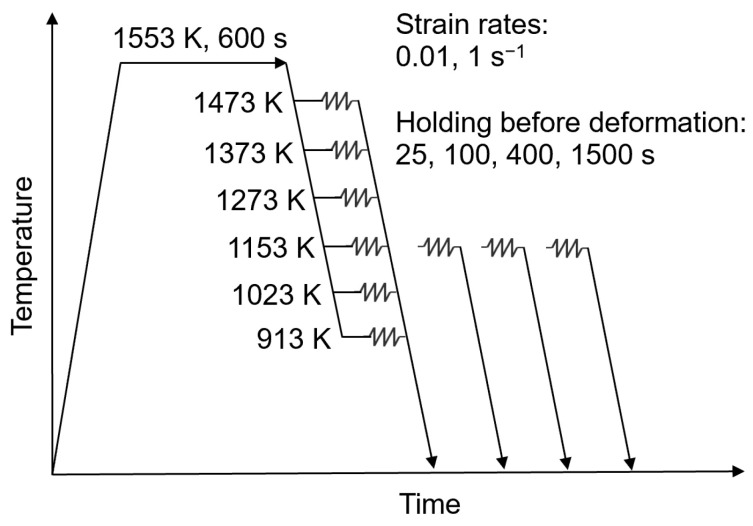
Thermo-mechanical rolling pattern for performed single-hit compression tests.

The strain-dependent development of the true stress as a function of temperature, intermediate holding time, and strain rate was investigated experimentally via uniaxial isothermal single-hit compression tests on a Gleeble^®^ 3800 thermo-mechanical simulator. Cylindrical samples of the microalloyed steels with 10 mm diameter and 15 mm length were tested. Figure 3 depicts the performed thermo-mechanical treatments schematically. An R-type thermocouple was welded to the surface of the specimen in order to control the temperature during the experiment. Additionally, a sandwich of Mo, nickel paste, and graphite was placed between the sample and the anvil to decrease the temperature gradient. Preliminary tests using one thermocouple welded at the center and the border of the sample showed a temperature difference smaller than 10 K.

All samples were solution annealed at 1553 K for 600 s. After cooling down to the corresponding test temperature (1473–913 K) with 70 Ks^−1^, the samples were held for 25 s before the compression to a true strain of 1 with 0.01 s^−1^ strain rate. Additional tests were performed at 1153 K with extended holding times of 100–1500 s before compression at a strain rate of 1 s^−1^. The higher strain rate assures the applicability of our findings to real industrial rolling schedules. Tantalum foils and nickel paste were placed between the sample and the anvil to reduce friction. During the tests, a high-vacuum atmosphere was created to avoid the oxidation of the surface at elevated temperatures. Each experiment was carried out twice for reproducible accuracy.

For an exact determination of experimental flow curves without contributions of, e.g., machine deformation and rigidity, special attention must be laid upon the detected length signal. The impact of the machine stiffness on the effective stress and strain in the framework of our experiments is discussed in Appendix A.

## 4. Results and Discussion

The experimental stress–strain curves and the derived *σ*_0_, *σ*_∞_, and *θ*_0_ were used for the evaluation of the strengthening coefficients *A*, *B*, and *C* that control the dislocation density evolution. The calculated ABC strengthening parameters, in turn, serve as input for the modified Kocks–Mecking model, which was applied to reproduce the experimental flow curves.

### 4.1. Experimental Flow Curves

The flow curves show the relationship between the applied true stress *σ* and the true strain *φ* for the plastic deformation range of the microalloyed steel grades.

#### 4.1.1. Steel Grades S1–S5

**Figure 4:**
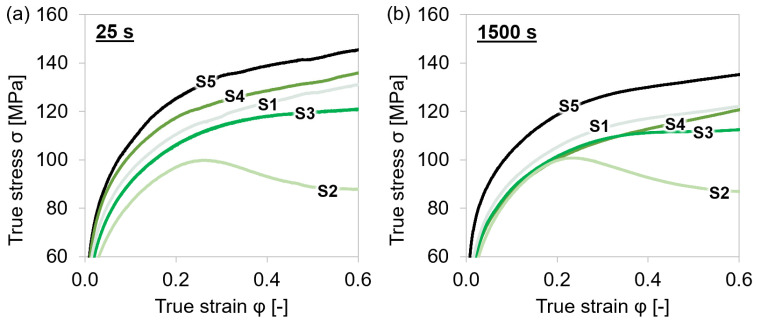
Stress–strain curves during single-hit compression tests with varying holding times before deformation and 0.01 s^−1^ strain rate at 1153 K for MA steel grades S1–S5. (**a**) 25 s; (**b**) 1500 s.

Figure 4 depicts the stress behavior of all microalloyed steels tested at 1153 K after different holding times before deformation. The graphs indicate that the alloy S5, with the combination of the highest content of Nb and V, reached the highest true stress values followed by the other alloys in the sequence S4 (Nb alloyed)/S1 (slight Nb alloying with Ti and V), S3 (V-alloyed), and S2 (no Ti, Nb, V microalloying), independent of holding time. The stresses of S4 exceeded the values of the 0.1 wt % V-alloyed S3, which showed that the strengthening effect of 0.03 wt % Nb was more effective than the one provided by V. The stress of steel grade S5 was enhanced the most due to higher amounts of (Ti,Nb,V)(C,N) precipitates. Only the least alloyed S2 steel showed softening at a true strain of approximately 0.25 caused by dynamic recrystallization. As S2 contains the lowest amounts of Ti, V, and Nb, the start temperature for recrystallization was expected to be lower than for the other materials as less (Ti,Nb,V)(C,N) were present for the impediment of the grain boundary movement. In the case of dynamic recrystallization at high temperatures, not only pinning effects on the dislocations but also pinning on the grain boundary should be considered.

Except for S2, the true stresses of the materials reached higher values after shorter holding at the test temperature. This result can be explained by the impact of microalloy carbonitrides on strengthening and is discussed in detail in Section 4.4.3.

#### 4.1.2. Variation of Test Temperatures, Intermediate Holding Times, and Strain Rates

The steel grade S5 was only examined at 1153 K, in order to analyze the effect of high microalloying and different precipitation sequences on the strengthening.

**Figure 5:**
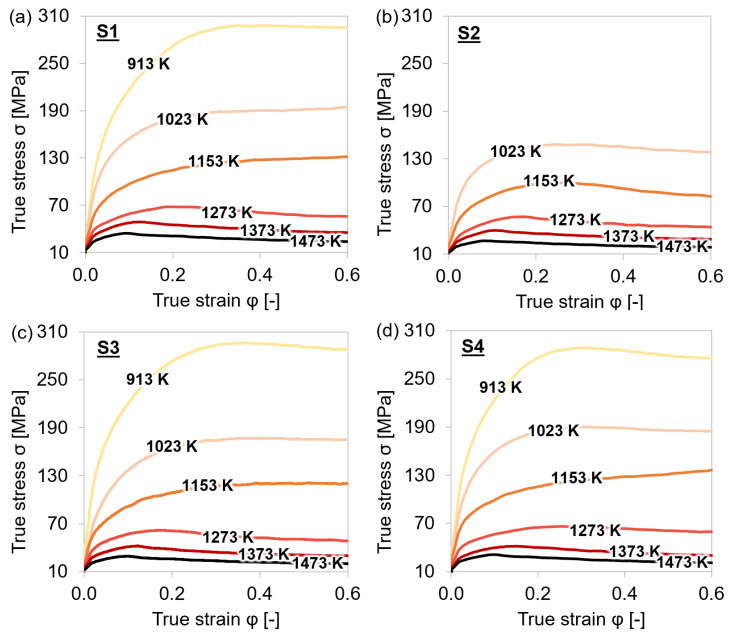
Stress–strain curves during single-hit compression tests with 25 s holding time before deformation and 0.01 s^−1^ strain rate at 913–1153 K. (**a**–**d**) MA steel grades S1–S4.

Figure 5 illustrates the temperature dependence of the stresses of the individual steel grades S1–S4. At elevated temperatures, softening of the material was observed at lower strains due to higher-grain boundary mobilities. The presence of peak stresses indicated that dynamic recrystallization had already started. The higher the temperature, the lower the true stress values, as recovery and recrystallization processes lead to a reduction of the dislocation density.

**Figure 6:**
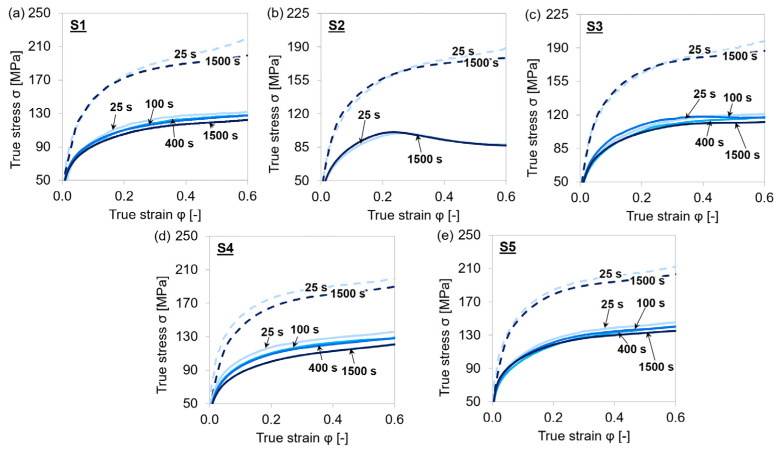
Stress–strain curves during single-hit compression tests with 25–1500 s holding times before deformation and 0.01 s^−1^ (continuous line) as well as 1 s^−1^ (dashed line) strain rates at 1153 K. (**a**–**e**) MA steel grades S1–S5.

Figure 6 depicts the stress–strain curves for each material with varying holding times before deformation and at two different strain rates. With higher strain rates, there was less time available for recovery and recrystallization processes, and therefore the stress increased significantly with increasing strain rate. The true stress slightly decreased when the material was held longer at the deformation temperature before plastic deformation. The prolonged holding may lead to coarsening of (Ti,Nb,V)(C,N) and agglomeration of the former finely dispersed precipitates as well as static recrystallization. These effects are expected to be more pronounced at the steel grades with higher amounts of microalloying elements such as S4 and S5.

The minuted influence of different holding times on the mechanical properties might appear unexpected, especially the tendency for lower stress levels at longer holding times. However, the observed behavior indicated that the precipitation process is rather quick and has already reached overaging after holding for 25 s. It is also remarkably interesting that the precipitation sequence did not have a strong effect on the overall stress–strain behavior for any of the five materials. As a consequence, we did not expect a significant impact of the precipitation state on the overall dislocation density evolution.

### 4.2. Physical Parameters *σ*_0_, *σ*_∞_, and *θ*_0_

The previously discussed experimental stress–strain curves are used to derive *σ*_0_, *θ*_0_, and *σ*_∞_. The exact procedure for the determination of these physical parameters, as well as the linear approximations of the used Kocks plots, are given in Appendix B.

**Figure 7:**
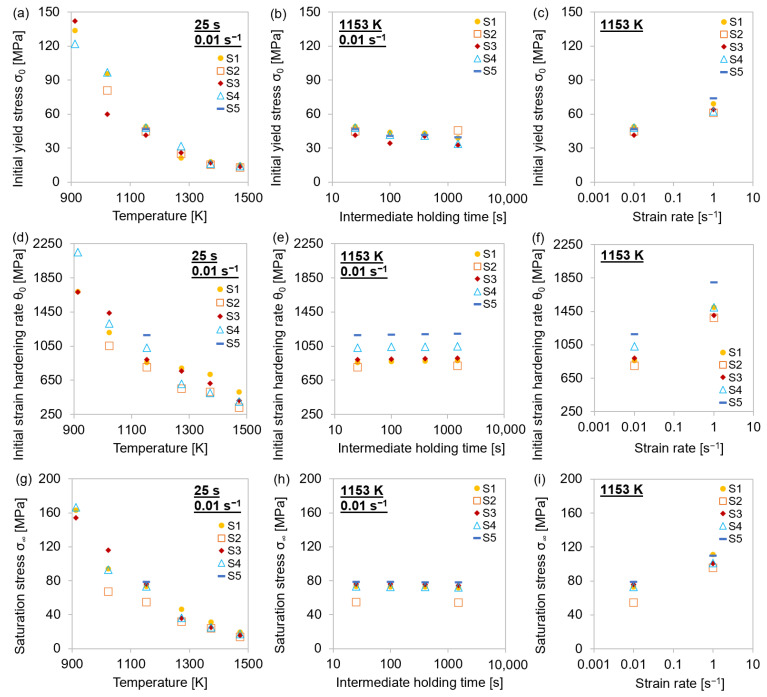
Physical parameters for single-hit compression tests with 25–1500 s holding times before deformation and 0.01–1 s^−1^ strain rates at 913–1473 K for MA steel grades S1–S5. (**a**–**c**) Initial yield stress *σ*_0_; (**d**–**f**) initial strain hardening rate *θ*_0_; (**g**–**i**) saturation stress *σ*_∞_.

Figure 7 shows the determined *σ*_0_, *θ*_0_, and *σ*_∞_ for the different thermo-mechanical treatments.

**Figure 8:**
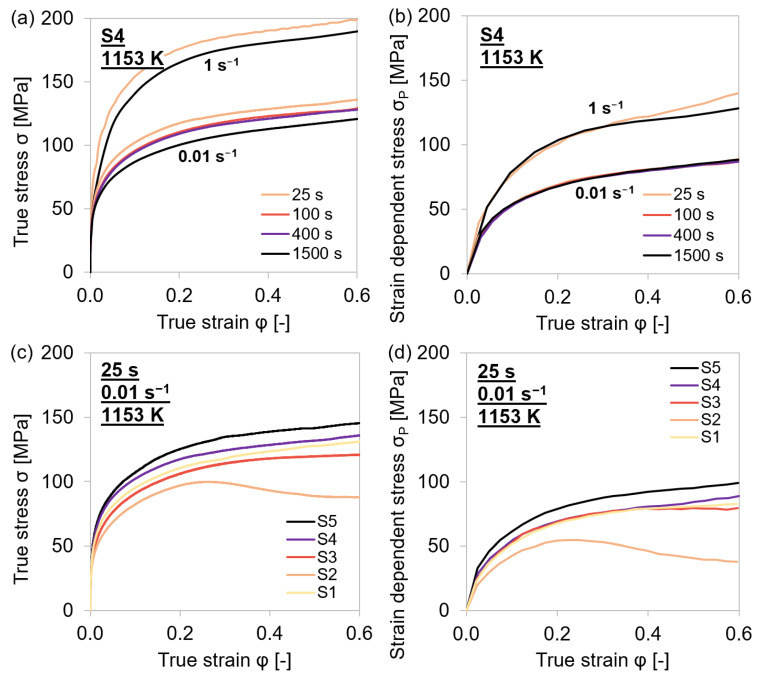
Comparison of experimental stress–strain and flow curves during single-hit compression tests with 25–1500 s holding times before deformation and 0.01–1 s^−1^ strain rates at 1153 K of MA steel grades S1–S5. (**a**,**c**) Entire stress–strain curves. (**b**,**d**) Flow curves for strain hardening area III.

It was observed that temperature and strain rate affected all three parameters proportionally. The values of *σ*_0_, *θ*_0_, and *σ*_∞_ decreased monotonically with temperature and increase with the strain rate. As for lower *θ*_0_ at elevated temperatures (Figure 7d), it can be stated that it took more deformation to reach *σ*_∞_ than at lower temperatures. The high strain hardening rates at lower temperatures, on the other hand, led to a high stress increase with compression. As indicated in Section 4.1.2, the influence of the holding time before the deformation was only shown to have a slight effect on the yield stress. That *σ*_0_ was the only stress that was affected by different holding times can be assured in a direct comparison of the entire stress–strain curves (Figure 8a,c) and the flow curve of *σ*_P_ against the true strain (Figure 8b,d), where the yield stress *σ*_0_ was subtracted from the rest of the stress–strain curve. Figure 8a,b shows such plots for different holding times of the same material, while Figure 8c,d shows those for the same holding time of different materials.

It became obvious that while the difference in the stress–strain curves for different holding times mainly stemmed from a difference in *σ*_0_—i.e., the curves in Figure 8b almost perfectly overlap—a change in the material had a much more pronounced influence on the values of *θ*_0_ and *σ*_∞_.

### 4.3. Strengthening Parameters A, B, and C

**Figure 9:**
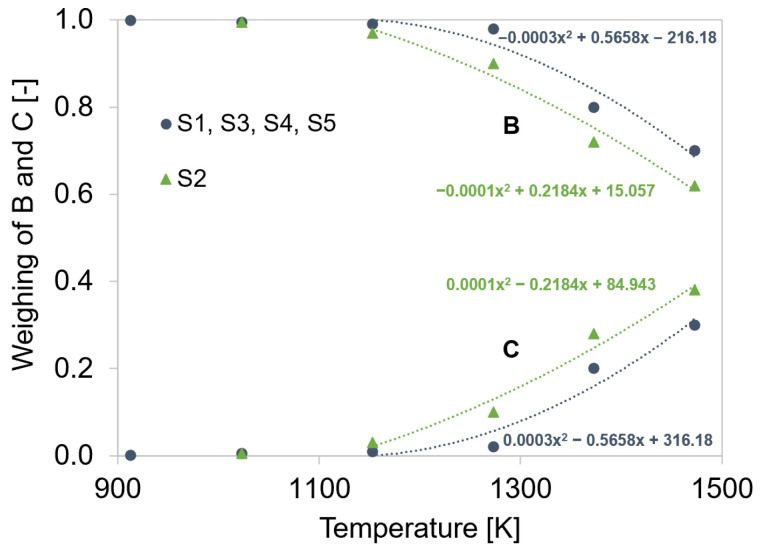
Temperature-dependent experimental weighing factor for parameters B and C.

The values for the strengthening coefficients *A*, *B*, and *C* were calculated via Equations (7)–(9), respectively. *B* and *C* had to be additionally multiplied with an experimental weighting factor, given in Figure 9, as the overall effect of recovery was divided into dynamic (B) and static (C) parts. At lower temperatures, *B* represented the dominant part, whereas, at elevated temperatures, *C* gained significance. The diffusion coefficient *D*_d_ increased exponentially with temperature (Equation (11)), which explains why the term *C* gained significance over the term *B* at elevated test temperatures.

It was experimentally determined that static recovery, and therefore *C*, increased above 1153 K. As the *C* parameter rose, climbing of dislocations to overcome obstacles was facilitated. The weighing of the *B* and *C* parameters in the intermediate temperature range was conducted to reproduce the best possible experimental curves with the simulation. The parameter *C* was negligible at low temperatures and high contents of microalloying elements. Microalloying elements possess the ability to retard recovery due to the formation of carbonitrides at dislocations. Steel grade S2 contains only negligible amounts of Ti, Nb, and V. Therefore, the mechanism of static recovery was more pronounced.

**Figure 10:**
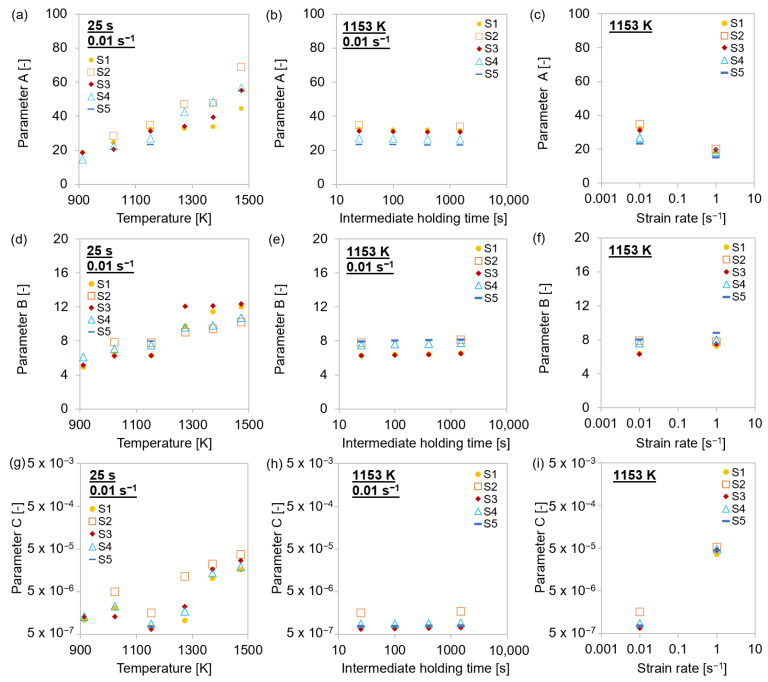
Strengthening parameters for single-hit compression tests with 25–1500 s holding times before deformation and 0.01–1 s^−1^ strain rates at 913–1473 K for MA steel grades S1–S5. (**a**–**c**) Parameter A; (**d**–**f**) parameter B; (**g**–**i**) parameter *C*.

Figure 10 shows that the coefficients *A*, *B*, and *C* increased at elevated temperatures, while the different intermediate holding times showed no impact on the values. Additionally, the ABC parameters were deformation rate dependent.

### 4.4. Computational Analysis of the Results

Thermokinetic simulation concerning the influence of dislocation density evolution on the mechanical steel properties was performed using the ABC-model with evaluated parameter values as described in the previous section. The effect of precipitated MX phases on the strengthening behavior of the examined steel grades was analyzed via a comparison of the simulated yield strength contribution from precipitates to the experimental results. In this chapter, the dislocation density evolution and all flow curves are modeled and treated independently with their respective individual-experiment-based parameters.

#### 4.4.1. Dislocation Density Evolution

**Figure 11:**
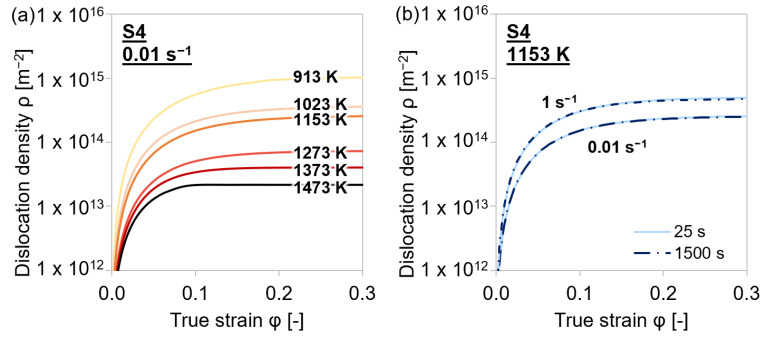
Simulation results of dislocation density evolution during single-hit compression tests with varying holding times before deformation, strain rates, and temperatures of MA steel grade S4. (**a**) 25 s, 0.01 s^−1^, 913–1473 K; (**b**) 25–1500 s, 0.01–1 s^−1^, 1153 K.

Figure 11 illustrates that the dislocation densities, evaluated according to Equation (6), reached higher values at decreasing temperatures and faster strain rates. The variation of the intermediate holding time had no impact on the dislocation density. The higher dislocation density at lower temperatures was expected to provide more nucleation sites for (Ti,V)(C,N) precipitates, leading to an increase in strain hardening. At elevated temperatures, the dislocation density decreased due to accelerated annihilation.

#### 4.4.2. Flow Curves

**Figure 12:**
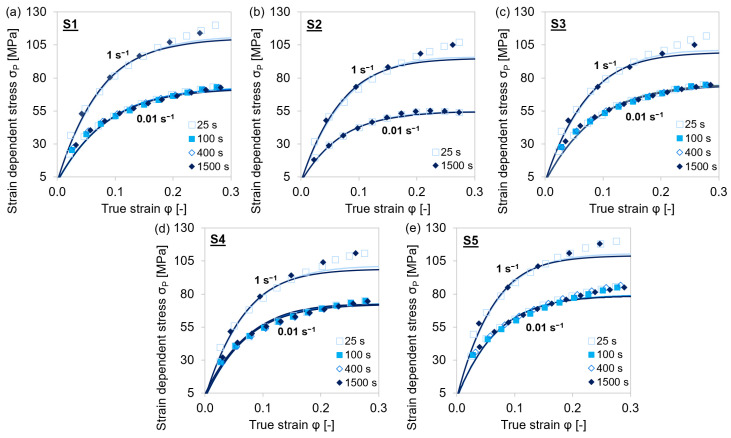
Comparison of experimental *σ*_P_ (symbols) and simulated (lines) flow curves during single-hit compression tests with 25–1500 s holding times before deformation and 0.01–1 s^−1^ strain rates at 1153 K. (**a**–**e**) MA steels S1–S5.

**Figure 13:**
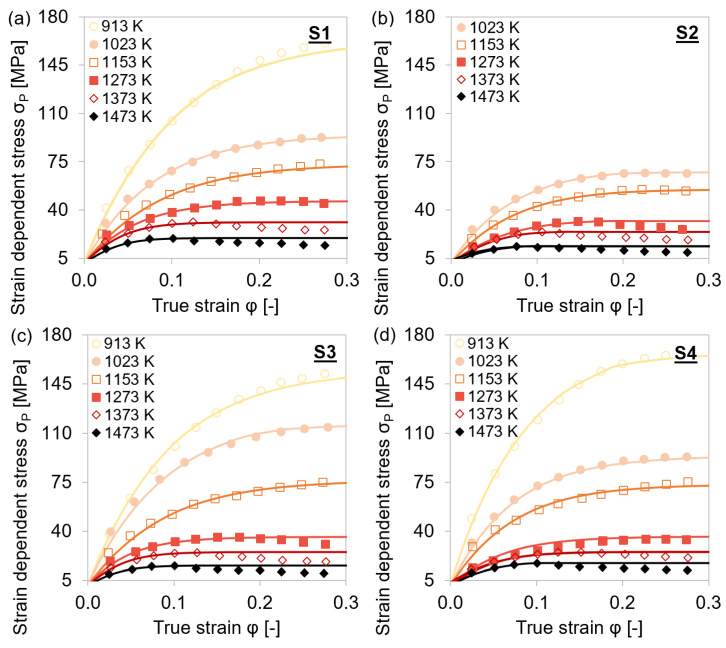
Comparison of experimental *σ*_P_ (symbols) and simulated (lines) flow curves during single-hit compression tests with 25 s holding time before deformation, 0.01 s^−1^ strain rate at 913–1473 K. (**a**–**d**) MA steels S1–S4.

The simulative dislocation strength contribution in the precipitation domain austenite was compared to the experimental-strain-dependent and dislocation-density-affected plastic stress *σ*_P_ = *σ* − *σ*_0_ (Figure 12 and Figure 13). The applied extended Kocks–Mecking model was limited to the region of dynamic recovery and did not account for dynamic recrystallization at true stresses above the critical strain (≈0.3).

The experimental stress–strain values were accurately reproduced by the results of the simulation via the MatCalc ABC-model for the dislocation density evolution. Slight deviations at higher strain and lower temperatures were caused by dynamic recrystallization of the material. This phenomenon was not taken into account in the model. At alloy S2, which did not contain significant amounts of microalloying elements, recrystallization and therefore the deviation from the experiments was more pronounced.

#### 4.4.3. Impact of Microalloy Carbonitrides on Strengthening

Investigations of the experimental total yield strength (Figure 5 and Figure 6) showed that the stress decreased at higher temperatures, lower deformation rates, and extended holding before the deformation.

**Figure 14:**
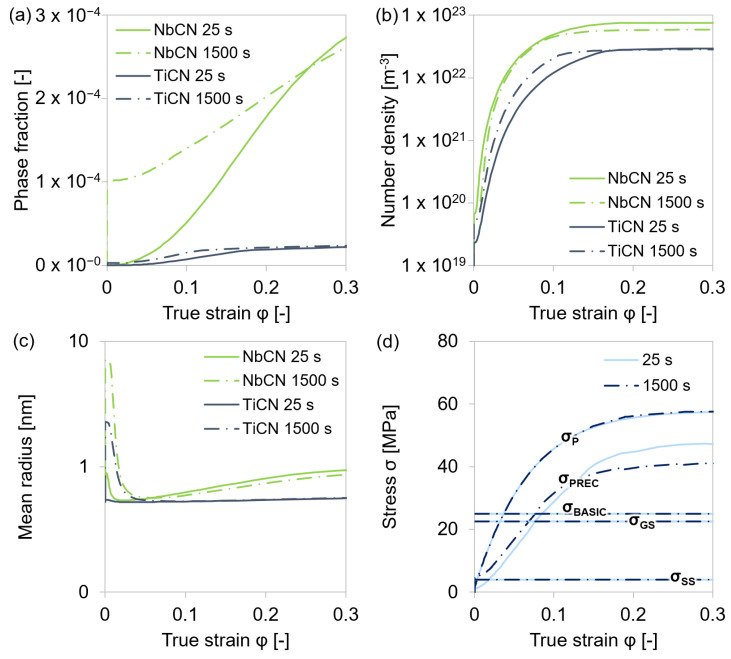
Analysis of precipitates and stresses during simulation of a single-hit compression test of steel grade S4 at 1153 K with varied holding time before deformation and 0.01 s^−1^ strain rate. (**a**) Phase fraction of precipitates. (**b**) Number density of precipitates. (**c**) Mean radius of precipitates. (**d**) Stress contributions without thermal activation of simulated stress–strain curves with ABC parameters calculated from Equations (7)–(9).

To examine the role of precipitates on this strengthening behavior, the particle distributions, as well as the individual, attained stress contributions from compression tests of steel grade S4 with 0.01 s^−1^ strain rate to a true strain of 1 after 25 s and 1500 s holding at 1553 K (Figure 3) were analyzed via MatCalc simulation (Figure 14).

At the exemplary examined alloy S4, the phase fractions, number densities, and mean radii of (Ti/Nb)(C,N) precipitates increased during deformation (Figure 14a–c). Due to the higher amount of microalloyed niobium, Nb(C,N) predominated. Longer holding at the test temperature caused a higher phase fraction of the particles before deformation started, but shorter holding times before deformation, resulted in a higher phase fraction, number density, and mean radius of the particles at the end of the compression. Therefore, shorter holding at the respective test temperature caused an accelerated formation of precipitates during deformation. These results are in good accordance with the simulated stress contributions. Figure 14d shows the strain-dependent stress *σ*_P_ as well as the stress contributions to the mechanical threshold $\hat{\sigma }$ according to Equation (17). It appears that a varied holding time before the deformation of the material only had an impact on *σ*_PREC_. Shorter holding caused higher stress values, which can be accounted entirely to precipitates.

In Section 4.1.1., it is discussed that only the least alloyed steel S2 showed softening at a true strain >0.2 caused by dynamic recrystallization. The mentioned alloy contained the lowest amounts of microalloying elements, and therefore the start temperature for recrystallization impediment due to formed carbonitrides was expected to be lower than for the other alloys. The test temperature of 1153 K was assumed to be too low for dynamic recrystallization of the alloys S1 and S3–S5, and therefore deformation-induced recrystallization had no impact on *σ*_GS_ and *σ*_P_. Austenite grain growth generally takes place during annealing treatments at high temperatures (1553 K in this work). At 1153 K, hardly any grain growth was expected during prolonged holding. As the grain boundary strength contribution is mainly controlled by the grain size, it is reasonable that the holding time did not influence *σ*_GS_ at 1153 K. The solid solution strength contribution of ≈4 MPa was rather small in comparison to the other stress contributions. The decrease in solute microalloying elements due to the deformation-induced precipitation of microalloy carbonitrides apparently had no impact on *σ*_SS_.

**Figure 15:**
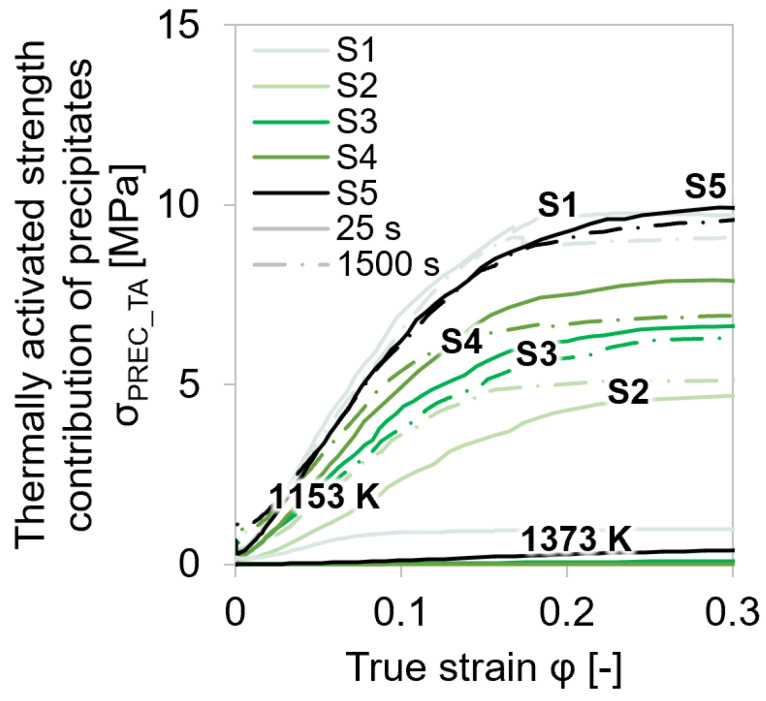
Comparison of thermally activated strength contribution from precipitates at varied temperatures and holding times before single-hit deformation for steel grades S1–S5.

Figure 15 compares the thermally activated strength contribution from precipitates at two different temperatures and at two different holding times before the deformation of all five examined materials.

*σ*_PREC_TA_ describes the temperature-dependent interplay of the dislocation motion with the precipitates, which acted as obstacles. At lower temperatures (1153 K), the thermally activated strength contribution of the precipitates increased as more and more carbonitride phases were formed, according to the equilibrium calculations shown in Figure 2. Furthermore, at higher temperatures (1373 K), the precipitates were mainly dissolved. In the studied microalloyed steel, *σ*_PREC_TA_ was very low because at elevated temperatures, the dislocations can climb through the precipitates with much less stress requirement [72]. It can be concluded that precipitates had negligible influence on the total stresses, and therefore, on the dislocation evolution. Thus, the experimental determination of *σ*_0_ and subsequently *σ*_P_ can be realized via the presented method in Section 1.1.

The simulation confirms that carbonitride precipitates mainly form on dislocations—and not in the bulk—as the simulated increase in precipitation hardening, with nucleation sites selected as dislocations coincided with the experimental strength gain from varied holding before deformation.

### 4.5. Description of ABC Parameters by Newly Developed Equations

The experimental investigations of stress–strain curves with different intermediate holding times, temperatures, strain rates, and microalloyed steel grades showed that the dislocation density evolution was mainly governed by temperature and strain rate changes and was only secondarily dependent on the composition of the material. This allows one to establish a single set of temperature- and strain-rate-dependent *A*, *B*, and *C* parameters averaged over all microalloyed steels.

It is applicable for various microalloyed steel grades in a wide temperature range. Table 3 lists the according equations for *A*, *B*, and *C*, derived from the average parameters of all five steel grades in a temperature range of 913–1473 K and 0.01–1 s^−1^ strain rate. When applying the following equations, an initial dislocation density *ρ_eq_* of 1∙10^11^ was used.

**Figure 16:**
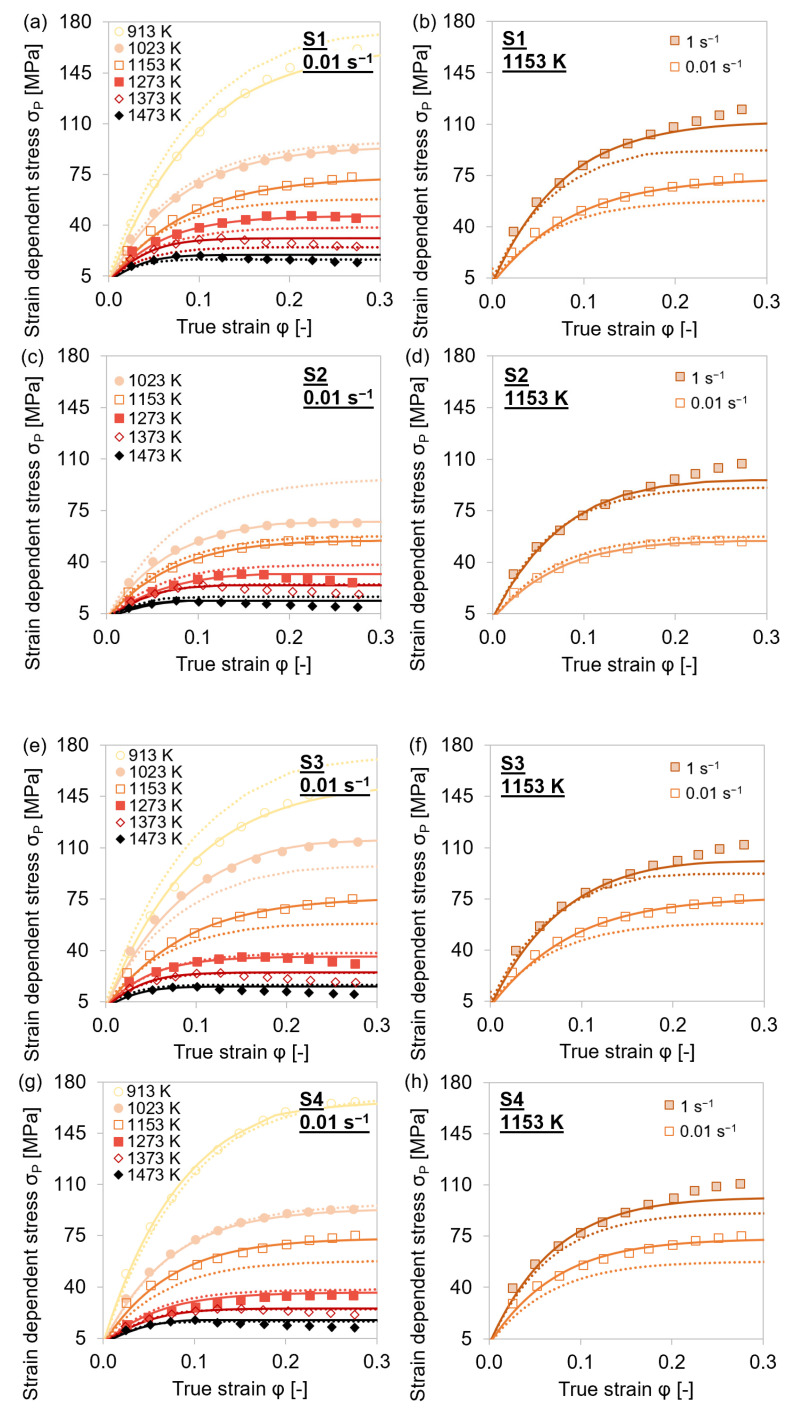
Comparison of experimental (symbols), simulated with experimentally determined ABC parameters (lines), and simulated with ABC parameters calculated from equation (dotted lines) flow curves during single-hit compression tests with 25 s holding time before deformation of steel grades S1–S4. (**a**,**c**,**e**,**g**) T = 913–1473 K, $\dot{\varphi }$ = 0.01 s^−1^; (**b**,**d**,**f**,**h**) T = 1153 K, $\dot{\varphi }$ = 0.01–1 s^−1^.

Figure 16 compares the experimental flow curves as well as the simulated flow curves derived from experimentally determined *σ*_0_, *θ*_0_, and *σ*_∞_ for the specific steel grades, temperature, and strain rate combination with those resulting from the averaged ABC parameters. The depicted flow curves exemplarily show that the equations can also be applied for slight microalloying as the steel grades S3 and S4 only comprise Nb or V, respectively. The reference alloy S1 contains all three microalloying elements Ti, Nb, and V, plus a notable higher amount of Cr. Steel grade S2 served as a reference due to the low microalloying. In principle, the equations represent a suitable base for the computation of flow curves in microalloyed steel, which further implies that the associated estimation of the dislocation density evolution is within the order of magnitude reported in the literature for similar materials under similar conditions [16,73].

## 5. Summary and Conclusions

Isothermal single-hit compression tests of five Ti, Nb, and V microalloyed steel grades were employed for the simulative analysis of microstructure strengthening and the associated dislocation density development.

Originating from the experimental stress–strain curves, the initial yield stress *σ*_0_, the saturation stress *σ*_∞_, and the initial strain hardening rate *θ*_0_ were determined for different variations of thermo-mechanical treatments and different chemical compositions. The assessment of the parameters revealed a strong temperature and strain rate dependence, while the impact of varying holding times before deformation was negligible. The values of *σ*_0_, *σ*_∞_, and *θ*_0_ were used for the calculation of the ABC parameters, which served as physical model input for the extended mean-field Kocks–Mecking model for the dislocation density evolution. This model was then used to reproduce the experimental flow curves. The simulated dislocation density evolution as well as the true stress–true strain curves of microalloyed steel grades with varying Ti, Nb, and V agreed with the experimental trends. In the case of stress increase at faster deformation and lower studied temperatures, the dislocation density evolution strongly affected the stress–strain behavior. Strain hardening was increased at higher strain rates, decreasing temperatures, and higher contents of microalloying elements. At higher temperatures, static recovery becomes more relevant.

The presented semi-empirical state-parameter model allows for the description of the strain-induced density evolution of isolated dislocations. The calculated dislocation densities were in the same order of magnitude as those in the literature. The model can be applied for the calculation of flow curves of non-tested deformation conditions.

As the realistic prediction of the dislocation density evolution is not only relevant for strain hardening but also affects other processes such as recrystallization, our findings can be used as input for a wide range of microstructure simulations. The described dislocation density evolution also induces dynamic nucleation of (Ti,Nb,V)(C,N) carbonitrides and therefore has an impact on the final mechanical properties. Equations for the estimation of *A*, *B*, and *C* were introduced to establish an averaged ABC input dataset for simulations of various microalloyed steels. These can simplify the usability of the dislocation density model for real technological applications. The high-temperature dislocation density model with our new parameterization can be applied for different microalloyed steel grades and precipitation states. The simulation results demonstrated that the new equations are a promising tool for an average assessment of the strengthening parameters for different variations of microalloying.

## Figures and Tables

**Table 1:** Chemical compositions of the investigated materials in wt %. Balance Fe.

Steel Grade	C	Mn	Ti	Nb	V	Cr
S1	0.15	1.27	0.01	0.01	0.07	0.20
S2	0.18	0.88	<0.01	<0.01	<0.01	0.02
S3	0.20	1.40	<0.01	<0.01	0.10	0.02
S4	0.18	0.93	<0.01	0.03	<0.01	0.03
S5	0.21	1.45	<0.01	0.04	0.10	0.03

**Table 2:** Parameter symbols and values used for the ABC-model.

Symbol	Name	Value	Unit	Literature Source
ν	Poisson’s ratio	0.30	-	[68]
G	Shear modulus	$\frac{193000\;-\;73.33\cdot T\left[{}^{\circ}C\right]}{2\left(1\;+\;\nu \right)}$	MPa	MatCalc default value
b	Burgers vector	2.50∙10^−10^	m	[69]
M	Taylor factor	3.06	-	[69]
k	Boltzmann constant	1.38065∙10^−23^	JK^−1^	[70]

**Table 3:** Equations for the calculation of the ABC parameters as a function of temperature and strain rate, optimized for T = 913–1473 K and $\dot{\varphi }$ = 0.01–1 s^−1^, for the average of all investigated alloys.

**A for 913–1473 K**
$A=\left(6.48\cdot 10^{-2}\cdot \frac{T}{\left[K\right]}-43.10\right)+\left(7.96\cdot \frac{\dot{\varphi }}{\left[s^{-1}\right]}-20.68\right)\left(\frac{\dot{\varphi }}{\left[s^{-1}\right]}-0.01\right)$
**B for 913–1473 K**
$B=\left(1.11\cdot 10^{-2}\cdot \frac{T}{\left[K\right]}-4.70\right)+\left(5.02\cdot 10^{-2}\cdot \frac{\dot{\varphi }}{\left[s^{-1}\right]}+0.51\right)\left(\frac{\dot{\varphi }}{\left[s^{-1}\right]}-0.01\right)$
**C for 1273–1473 K**
$C_{HT}=\left(1.05\cdot 10^{-7}\cdot \frac{T}{\left[K\right]}-1.29\cdot 10^{-4}\right)+\left(4.69\cdot 10^{-5}\cdot \frac{\dot{\varphi }}{\left[s^{-1}\right]}+4.99\cdot 10^{-7}\right)\left(\frac{\dot{\varphi }}{\left[s^{-1}\right]}-0.01\right)$
**C for 913–1273 K**
$C_{LT}=\left(5.60\cdot 10^{-9}\cdot \frac{T}{\left[K\right]}-3.86\cdot 10^{-6}\right)+\left(4.69\cdot 10^{-5}\cdot \frac{\dot{\varphi }}{\left[s^{-1}\right]}+4.99\cdot 10^{-7}\right)\left(\frac{\dot{\varphi }}{\left[s^{-1}\right]}-0.01\right)$

**Figure A1:**
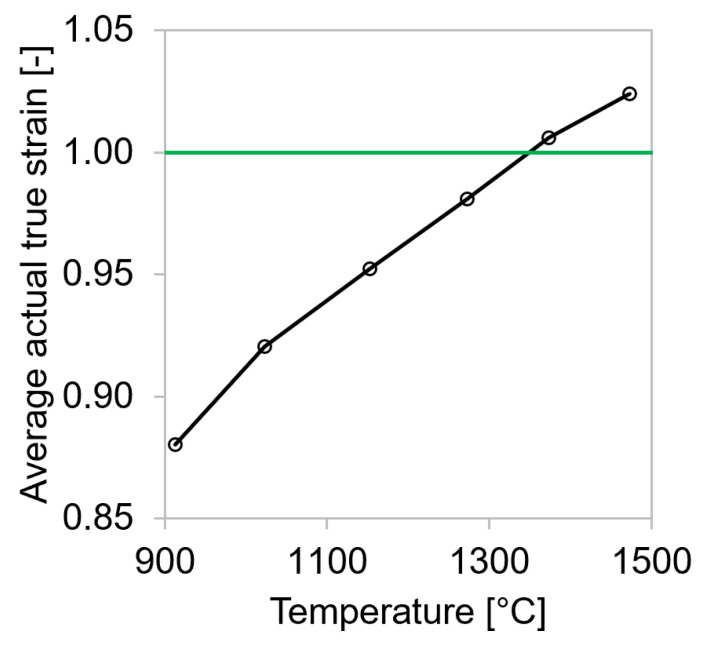
Increase in the average actual true strain with rising deformation temperature of single-hit compression tests with 25 s holding time before deformation and 0.01 s^−1^ strain rate at 913–1473 K for MA steel grades S1–S5.

**Figure A2:**
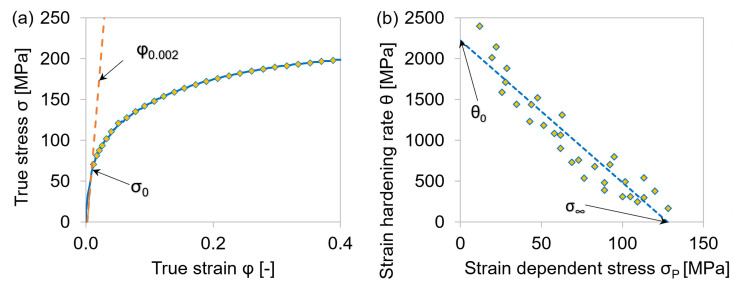
(**a**) Exemplary stress–strain curve with smoothed values; (**b**) linear fit of corresponding Kocks plot.

**Figure A3:**
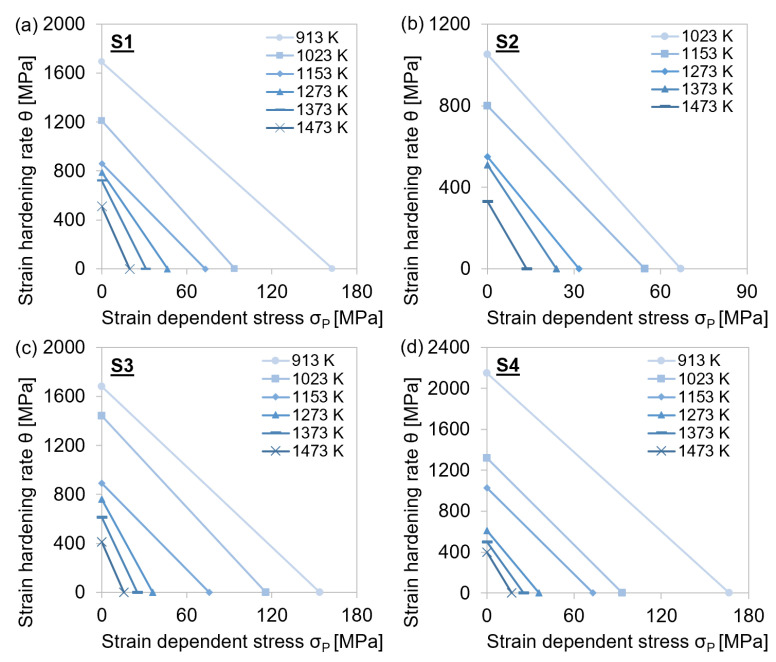
Linear fits of Kocks plots for single-hit compression tests with 25 s holding time before deformation and 0.01 s^−1^ strain rate at 913–1473 K. (**a**–**d**) MA steel grades S1–S4.

**Figure A4:**
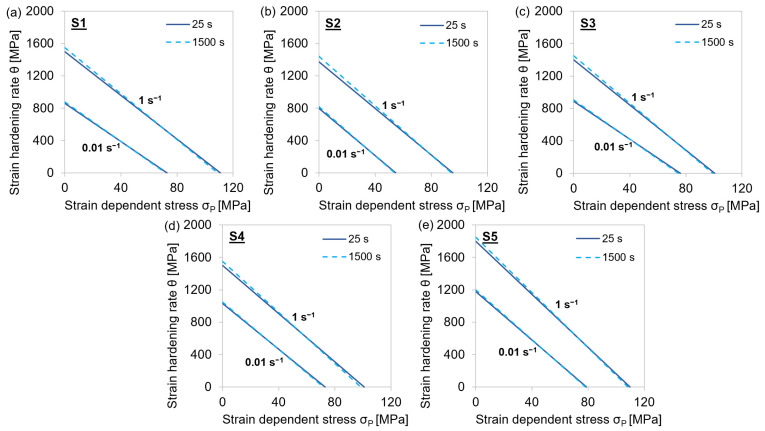
Linear fits of Kocks plots for single-hit compression tests with 25–1500 s holding times before deformation and 0.01 s^−1^ strain rate at 1153 K. (**a**–**e**) MA steel grades S1–S5.

## Data Availability

Not applicable.

## Appendix A. Consideration of the Stiffness of the Gleeble^®^ Device in the Calculation of the Flow Curves

The sample length is measured as length change ∆*L*(*t*), relative to the start value *L_0_*. The length of the specimen *L*(*t*) is given by

(A1) $$L\left(t\right)=L_{0}-\Delta L\left(t\right).$$

One simple way of compensating for the machine rigidity is by comparing the final measured signal *L*_1_ at *t*_1_ with the final length of the sample *L_m_* measured after testing. Therefore, the compensated length change is given by

(A2) $$L_{comp}\left(t\right)=L_{0}-\Delta L\left(t\right)\frac{L_{1}}{L_{m}}.$$

In this paper, *L_comp_*(*t*) is used for the calculation of any derived quantities, e.g., stress and strain. With this adaptation, the exact deformation and real stress–strain curves of materials can be assessed using the equations listed in the following:

(A3) $$\varphi =ln\frac{L_{0}}{L_{comp}\left(t\right)},$$

(A4) $$\sigma =\frac{F}{\frac{V_{0}}{L_{comp}\left(t\right)}},$$

where *F* describes the applied force and *V*_0_ is the sample volume before the compression. The length adjustments have to be performed for all experimental flow curves.

After the path signal adjustment of all flow curves, it can be stated that the deviation from the estimated true strain 1 changed with the deformation temperature (Figure A1). At elevated temperatures, the true strain reached higher values than 1, whereas compression at 913 K resulted in an average actual true strain for all materials of only 0.88. The impact of the machine stiffness was more severe at lower temperatures. A length compensation for the calculation of the accurate stress–strain curve is therefore highly recommended.

## Appendix B. Kocks Plots for Determination of *θ*_0_ and *σ*_∞_

The first step for the practical provision of the necessary physical material parameters for the extended Kocks–Mecking model is the determination of *σ*_0_ at *φ*_0.002_ (Figure A2a). The yield stress at deformation start *σ*_0_, also called proof stress, was determined at the intersection point of the stress–strain curve and the proportional line offset to 0.002 true strain. Although more physical approaches are provided in literature concerning the initiation of dislocation motion (e.g., by Arechabaleta et al. [74]), the well-established 0.002 strain offset method was applied for the determination of *σ*_0_ in order to assure comparability with previous findings on the basis of the same approach.

The attained flow curves—*σ*_P_ plotted against *φ*—only describe stage III hardening and start at 0 MPa for better clarity. The values for the strain hardening rate *θ* were calculated via numerical differentiation of *dσ*/*dφ*, and **σ*_P_* was equal to *σ*-*σ*_0_. The resulting Kocks plot from the graph of *θ* versus *σ*_P_ (Figure A2b) could then be used to read out *θ*_0_ and *σ*_∞_ for each individual curve.

The generated Kocks plots of the microalloyed materials showed that *θ*_0_ and *σ*_∞_ increased at decreasing temperatures (Figure A3). At longer holding times, *θ*_0_ showed a slight increase, while *σ*_∞_ decreased (Figure A4). However, these trends were insignificant, and constant values could be used for the simulation of flow curves concerning different intermediate holding times. The strain rate had a high impact on the initial strain hardening rate and the saturation stress. The graphs show this multiplicative correlation and reached higher values at fast strain rates, in accordance.
